# Healthcare workers safety in the COVID-19 era: the impact of pre-pandemic personal protective equipment (PPE) training in Pakistan

**DOI:** 10.1186/s12913-023-10048-y

**Published:** 2023-11-15

**Authors:** Zia Ul Haq, Zarghona Fazal Sher, Farhad Ali Khattak, Zala, Muhammad Hakim, Naeem Ullah, Abid Rahim, Umar Hussain, Saima Afaq

**Affiliations:** 1 Khyber Medical University, Peshawar, Pakistan; 2 Gandhara University, Peshawar, Pakistan; 3 Khyber College of Dentistry, Peshawar, Pakistan; 4 Saidu Medical College, Swat, Pakistan; 5 Saidu College of Dentistry, Swat, Pakistan; 6 Deptt of Epidemiology and Biostatistics, School of Public Health, Faculty of Medicine, Imperial College London, London, W2 1PG UK; 7 Department of Health Sciences, University of York, York, UK

**Keywords:** COVID-19, Pandemic, Personal protective equipment, Training, Pakistan, Healthcare Workers

## Abstract

**Background:**

The COVID-19 pandemic has revealed vulnerabilities in healthcare systems worldwide, emphasizing the importance of healthcare worker safety through adequate utilization of personal protective equipment (PPE). This study aims to assess the impact of pre-pandemic PPE training on the practices and other associated factors among frontline healthcare workers during the COVID-19 pandemic in Pakistan and provide insights into the implications of such training programs for future initiatives.

**Methods:**

A cross-sectional study from May 9th to June 5th, 2020 was conducted among the frontline healthcare workers against COVID-19 in Pakistan, utilizing an online structured questionnaire shared via WhatsApp and Facebook by using purposive sampling. Statistical analyses, including chi-square tests for proportion and logistic regression for the association while multi-logistic regression for potential confounders, were performed using SPSS version 22.

**Results:**

A total of 453 healthcare staff participated, with 68.9% (n = 312) reporting no prior PPE training and 31.1% (n = 141) having received training. Significant associations were found between prior training and healthcare group distribution (p = 0.006), with doctors exhibiting the highest proportion of training 82 (37.61%), followed by nurses 50 (27.32%) and paramedics 9 (17.31%). Those who didn’t receive any prior training in PPEs showed a higher perceived professional risk of 216 (69.23%) compared to those who received prior PPE training 96 (30.77%, p-value 0.005). Similarly, a higher frequency 137 (63.72%) of Perceived Personal risk was observed in those who didn’t receive training, labeled as “high risk” compared to those who were trained 78 (36.28%, P value 0.02). Multi-logistic regression analysis identified paramedics as 0.26 times less likely to have received prior PPE training (Adjusted OR 0.26, 95% CI 0.10–0.65, p = 0.01) compared to medical doctors. Healthcare workers in tertiary care hospitals were 0.46 times less likely to undergo PPE training (Adjusted OR 0.46, 95% CI 0.25–0.87,p = 0.01) compared to those working at COVID-19 facilities/hospitals/quarantine centers. Likewise, individuals who doffed disposable gowns [Adjusted OR 3.86, (95% CI, 1.23–12.08, p = 0.02] were 3.86 times more interested in getting prior training in PPE compared to those who don’t have skills to wear them.

**Conclusion:**

Our findings highlight that healthcare levels, type of healthcare, and doffing skills are important predictors of whether healthcare workers have taken prior training in PPE. These findings imply developing effective training programs for healthcare workers to ensure safety while providing care during pandemics like COVID-19.

**Supplementary Information:**

The online version contains supplementary material available at 10.1186/s12913-023-10048-y.

## Introduction

The COVID-19 pandemic has emerged as a major global health crisis, presenting unprecedented challenges with far-reaching consequences for societies worldwide [1]. In particular, low- and middle-income countries (LMICs) have struggled with the dual impact of the pandemic and economic recession, requiring effective strategies to navigate and cope with this complex situation [2]. Pakistan, classified as a low- and middle-income country with a Human Development Index value of 0.560 and a recent ranking of 152 out of 189 countries [3], has faced significant healthcare challenges over the past decades [4, 5]. The COVID-19 outbreak has further exacerbated the threats faced by Pakistan, given its vulnerable healthcare system and the rising number of communicable and non-communicable diseases [6]. This alarming situation has raised concerns, especially for frontline healthcare workers who are at an increased risk of contracting COVID-19 due to their close proximity to both COVID-19 and non-COVID-19 patients within healthcare facilities. Ensuring the safety of healthcare workers is crucial to avoid the discontinuation of essential medical services to patients and prevent the cross-spread of COVID-19. However, at the beginning of the pandemic, there were inconsistent and ambiguous guidelines for personal protective equipment across organizations and countries [7].

The well-being and safety of healthcare workers are of utmost importance, and the Centers for Disease Control and Prevention (CDC) have provided crucial recommendations to ensure their protection [8]. When caring for patients with COVID-19, the CDC strongly advises healthcare workers to use Personal Protective Equipment (PPE) [8]. Safeguarding frontline healthcare workers from infection is essential for effective outbreak response, and the proper utilization of personal protective equipment (PPE) plays a crucial role in ensuring their safety [9, 10]. Apart from PPE design factors, ensuring meticulous donning and doffing of it is a crucial measure in minimizing contamination among healthcare workers caring for patients with transmissible infectious diseases [11]. Lacking sufficient education and training regarding proper PPE utilization can adversely affect compliance with the PPE use recommendations [12]. Thus, providing comprehensive education to healthcare providers about PPE could potentially be effective in curbing the transmission of COVID-19. However, studies indicate that many frontline healthcare workers lack adequate knowledge regarding the correct use of PPE, particularly in emergency rooms, isolation wards, intensive care units, and laboratories dedicated to COVID-19 patients [13]. Given these challenges, the COVID-19 pandemic has underscored the critical importance of managing PPE effectively to protect healthcare workers and prevent further transmission of the virus.

In this study, we aim to assess the impact of pre-pandemic PPE training on healthcare workers’ practices in handling and using PPE during the COVID-19 pandemic. Specifically, we examine the ability of healthcare workers who have undergone pre-pandemic PPE training to correctly don and doff various types of PPEs. Our study aims to assess PPE practices and other associated factors between trained and untrained healthcare workers during the COVID-19 pandemic. This investigation informs the effectiveness of training programs and guides future initiatives. Identifying associated factors and training impact helps to design safer programs for healthcare workers and patients by enhancing pandemic preparedness and worker safety.

## Material & methods

The study was conducted as a cross-sectional survey from May 9th to June 5th, 2020. The questionnaire was administered to frontline healthcare workers (doctors, nurses, and paramedics) working against the COVID-19 pandemic in major provinces of Pakistan. Ethical approval was obtained from the Khyber Medical University Ethics Review Board (Dir/Ethics/KMU/2020/17). Due to the lockdown measures, data collection was conducted online using a structured questionnaire developed by the University of Bologna (Italy) and Harvard University, which had been previously used in Italy (www.liebertpub.com/doi/suppl/10.1089/hs.2020.0142). The questionnaire was translated from Italian to English and underwent expert review by infection control personnel from five COVID-19 tertiary care hospitals. In our study, frontline health workers were approached and recruited through peer invitations via social media platforms like WhatsApp and Facebook. Detailed study information was shared upon expressing interest, emphasizing data confidentiality, privacy, and feedback commitment. Informed consent was obtained at the questionnaire’s start through a ‘Yes’ confirmation. The survey was voluntary, and no financial or other rewards were offered for completing the survey’s questionnaire [14]. In addition to this, all the methods/procedures were performed in accordance with the relevant guidelines and regulations (Declaration of Helsinki). Inclusion criteria encompassed healthcare workers aged 21 years or older, holding a valid professional license/registration, and working actively during the COVID-19 emergency in Pakistan. The questionnaire covered various aspects, including the demographic characteristics of the respondents, distribution of healthcare groups, location, gender, age category, work experience, working healthcare level, training provider, days of training, mode of training, and ability to don and doff the PPE. Perceived professional and personal risk levels among healthcare staff were analyzed for their association with prior training on PPE. By using SPSS version 22 for Statistical analyses, the chi-square tests were used to determine the significance of the associations. The logistic regression analysis aimed to examine the covariate effects on the likelihood of healthcare workers having undergone prior training on Personal Protective Equipment (PPE). Adjusted odds ratios were estimated to assess the strength of these associations while accounting for the potential confounding variables and controlling for their influence on the model.

## Results

A total of 453 healthcare workers participated in this study. Among them, 68.9% (n = 312) had no prior training in PPE, while 31.1% (n = 141) had received prior training (Table 1). The distribution of the healthcare group was significantly associated with prior training in PPE (p = 0.006), with doctors having a higher proportion of prior training 82 (37.61%) compared to nurses 50 (27.32%) and paramedics 09 (17.31%). The province/territory was also significantly associated with prior training (p = 0.05), with the highest proportion of prior training reported in Sindh 12 (48.00) followed by Baluchistan 06 (40.0) as compared to Punjab, Federal, and Sindh. There was a higher proportion of males 124 (33.24, P value 0.04) having prior training compared to females. Working at a healthcare level was significantly associated with prior training (p = 0.03), with healthcare staff working at COVID-19 facility/hospital/quarantine centers 38 (43.7%) or at tertiary healthcare levels 66 (29.73%) having a higher proportion of prior training. The mode of training was found to be significantly associated with prior training (p < 0.001), with a higher proportion of healthcare staff receiving training through face-to-face mode 109 (100.00%) compared to online or no training.

**Table 1:** Demographic characteristics of respondents

Characteristic	Prior Training in PPE received by Health Staff		p-value
	No N = 312(68.9%)	Yes N = 141(31.1%)	
**Which healthcare group?**			0.006
* Doctor*	136 (62.39)	82 (37.61)	
* Nurse*	133 (72.68)	50 (27.32)	
* Paramedics*	43 (82.69)	9 (17.31)	
**Location**			0.05
* Khyber Pakhtunkhwa*	227(69.00)	102 (31.00)	
* Punjab*	43(69.35)	19 (30.65)	
* Sindh*	13(52.00)	12 (48.00)	
* Baluchistan*	09 (60.00)	06 (40.00)	
* Federal*	20 (90.9.00)	02(9.1)	
**Gender**			0.04
* Female*	63 (78.75)	17 (21.25)	
* Male*	249 (66.76)	124 (33.24)	
**Age category (in years)**			0.60
* 21–34*	212 (70.20)	90 (29.80)	
* 35–48*	83 (65.35)	44 (34.65)	
* 49–61*	17 (70.83)	7 (29.17)	
**Work experience (in years)**			0.24
* 1–11*	243(68.5)	112(31.5)	
* 12–22*	57(74.0)	20(26.0)	
* 23–33*	10(52.6)	09(47.4)	
* More than 33*	02(100)	0(0)	
**Working at which healthcare level?**			0.03
* COVID-19 facility/hospital/quarantine center, Emergency Operation Center ((National and Provincial)*	49(56.3)	38 (43.7)	
* Primary level*	27 (77.14)	8 (22.86)	
* Secondary level*	80 (73.39)	29 (26.61)	
* Tertiary level*	156 (70.27)	66 (29.73)	
**Working at a facility/ward dedicated to COVID-19 patients?**			0.60
* Both*	89 (70.63)	37 (29.37)	
* No*	95 (65.52)	50 (34.48)	
* Yes*	128 (70.33)	54 (29.67)	
**By whom Training was given?**			0.4
* Government*	71 (74.74)	24 (25.26)	
* INGO/NGO*	10 (76.92)	3 (23.08)	
* None*	207 (66.35)	105 (33.65)	
* World Health Organisation/UN*	24 (72.73)	9 (27.27)	
**Days of Training**			0.4
* 1 Day*	74 (76.29)	23 (23.71)	
* 2 Days*	6 (66.67)	3 (33.33)	
* 3 Days*	11 (78.57)	3 (21.43)	
* More than 3 days*	14 (66.67)	7 (33.33)	
* None*	207 (66.35)	105 (33.65)	
**Mode of training**			< 0.001
* Face-to-Face*	0 (0.00)	109 (100.00)	
* None*	312 (100.00)	0 (0.00)	
* Online*	0 (0.00)	32 (100.00)	
**Perceived professional risk for the next 30 days**			0.005
* High risk*	216 (69.23)	96 (30.77)	
* Low risk*	10 (41.67)	14 (58.33)	
* Medium risk*	83 (75.45)	27 (24.55)	
* No risk*	3 (42.86)	4 (57.14)	
**Perceived Personal risk for the next 30 days**			0.025
* High risk*	137 (63.72)	78 (36.28)	
* Low risk*	71 (74.74)	24 (25.26)	
* Medium risk*	99 (75.00)	33 (25.00)	
* No risk*	5 (45.45)	6 (54.55)	

Table 2 reports the self-reported perception of information received and the risk. We found that the health staff who received prior training on PPE had a significantly higher proportion 78 (57.78, p-value 0.001) of receiving adequate information and information was clear 109 (47.60, p-value 0.001) and complete 95 (49.74, p-value 0.001) and useful 110 (41.83, p-value 0.001) compared to those who had not received prior training. In terms of perceived professional risk for the next 30 days, a higher percentage of staff 216 (69.23, p-value 0.005) who had not received prior training perceived themselves to be at higher risk. Additionally, those participants who received prior training perceived themselves to be at low personal risk 24 (25.26, p-value 0.02) for the next 30 days.

**Table 2:** Self-reported perception about information received and the risk

Characteristic	Prior Training of PPE received by Healthcare Staff (Total;453)		p-value
	No N = 312(68.9%)	Yes N = 141(31.1%)	
**Have you received adequate information?**			< 0.001
* Always*	57 (42.22)	78 (57.78)	
* Never*	77 (92.77)	6 (7.23)	
* Rarely*	83 (82.18)	18 (17.82)	
* Sometimes*	95 (70.90)	39 (29.10)	
**Was the information you received about PPE clear?**			< 0.001
* Agree*	120 (52.40)	109 (47.60)	
* Disagree*	104 (90.43)	11 (9.57)	
* Not Sure*	88 (80.73)	21 (19.27)	
**Was the information you received about PPE complete?**			< 0.001
* Agree*	96 (50.26)	95 (49.74)	
* Disagree*	119 (82.07)	26 (17.93)	
* Not Sure*	97 (82.91)	20 (17.09)	
**Was the information you received about PPE useful?**			< 0.001
* Agree*	153 (58.17)	110 (41.83)	
* Disagree*	84 (86.60)	13 (13.40)	
* Not Sure*	75 (80.65)	18 (19.35)	

Table 3 asses the ability of health care staff to don and doff personal protective equipment after receiving prior training. The result showed that prior training significantly improved the ability of healthcare workers to don and doff the PPE. Of those who did not receive prior training 68 (82.93%, p-value 0.001) were unable to don the coverall suit compared to 223 (64.45%) of those who did receive prior training. Similarly, there was a significant difference in the ability to don disposable gown where 47(85.45%, P value 0.003) of those who didn’t receive prior training were unable to don-off the gown compared to 247(65.52%) of those who did receive prior training. The ability to doff disposable gowns showed a significant difference between those who received prior training.

**Table 3:** Ability of Donning and Doffing PPE by comparing those received Prior training

Characteristic		Prior Training of PPE received by Healthcare Staff (Total;453)		p-value
		No N = 312(68.9%)	Yes N = 141(31.1%)	
**Donning Cover all Suit**				0.001
* No*	68 (82.93)		14 (17.07)	
* Yes*	223 (64.45)		123 (35.55)	
* I am not sure*	21 (84.00)		4 (16.00)	
**Donning Disposable Gown**				0.003
* No*	47 (85.45)		8 (14.55)	
* Yes*	247 (65.52)		130 (34.48)	
* I am not sure*	18 (85.71)		3 (14.29)	
**Donning Protecting Googles**				0.07
* No*	56(77.8)		16(22.2)	
* Yes*	256 (67.19)		125 (32.81)	
**Donning Surgical Gloves**				0.02
* No*	27(87.1)		04(12.9)	
* Yes*	285 (67.54)		137 (32.46)	
**Donning Disposable Cover Shoes**				0.003
* No*	49(86.0)		08(14.0)	
* Yes*	263 (66.41)		133 (33.59)	
**Doffing Surgical Masks (N95/99)**				0.01
* No*	61(81.3)		14(18.7)	
* Yes*	251 (66.40)		127 (33.60)	
**Doffing Coverall Suit**				0.01
* No*	101(77.7)		29(22.3)	
* Yes*	211 (65.33)		112 (34.67)	
**Doffing Disposable Gown**				< 0.001
* No*	81(87.1)		12(12.9)	
* Yes*	231 (64.17)		129 (35.83)	
**Doffing Protecting Googles**				0.007
* No*	87(79.8)		22(20.2)	
* Yes*	225 (65.41)		119 (34.59)	
**Doffing Surgical Gloves**				0.065
* No*	48(80.0)		12(20.0)	
* Yes*	264 (67.18)		129 (32.82)	
**Doffing Disposable Cover Shoes**				0.001
* No*	78(83.0)		16(17.0)	
* Yes*	234 (65.18)		125 (34.82)	

The logistic regression analysis was run to look at the association of various predictors on whether healthcare workers had taken prior training in personal protective equipment (PPE). Our dependent variable was binary. The result of the univariate analysis shows that several factors were significantly associated with the outcome variable **(**Table 4**)**. These factors included gender, health care groups, working at the health care level, donning a coverall suit, donning a disposable gown, donning surgical gloves donning disposable cover shoes, and doffing surgical masks (N 95/99). All these had P values less than 0.05, except for donning protecting goggles which had a p-value of 0.07.

After adjustment for all these factors in a multivariate analysis in the form of an adjusted Odds ratio, only Doffing Disposable Gown, health care groups, and working at health care levels remained significant. Among healthcare care workings groups, Paramedics were 0.26 times less likely to have prior training in PPE taken [Adjusted OR 0.26, (95% CI, 0.10–0.65, p = 0.01] than medical doctors. While looking those working in tertiary care hospitals are 0.46 times less likely to take training in PPE [Adjusted OR 0.46, (95% CI, 0.25–0.87, p = 0.01] than those working in CoVID-19 (CoVID-19 facility/hospital/quarantine center, Emergency Operation center both National and Provincial levels. Similarly, individuals who doff disposable gowns [Adjusted OR 3.86, (95% CI, 1.23–12.08, p = 0.02] were 3.86 times more interested in getting prior training in PPE compared to those who don’t have skills to wear them.

**Table 4:** Logistic regression analysis of Prior Training of PPE Taken (No/Yes) with different factors(n = 453)

Characteristics	Categories	Unadjusted OR Model1	95% CI		P Value	Adjusted OR Model2	95% CI		P Value
			Lower	Upper			Lower	Upper	
Gender	**Reference value (Female)**								
	Male	1.84	1.03	3.28	0.03	1.72	0.87	3.37	0.11
Location	**Reference value (Khyber Pakhtunkhwa)**								
	Punjab	0.98	0.54	1.77	0.95	1.08	0.52	2.22	0.82
	Sindh	2.05	0.90	4.65	0.08	1.99	0.72	5.49	0.18
	Baluchistan	1.48	0.51	4.27	0.46	0.55	0.16	1.92	0.35
	Federal	0.22	0.05	0.970	0.04	0.21	0.04	0.98	0.04
Which healthcare group?	**Reference value (Doctor)**								
	* Nurse*	0.62	0.40	0.95	0.029	0.67	0.40	1.12	0.12
	* Paramedics*	0.34	0.16	0.75	0.007	0.26	0.10	0.658	0.01
Working at which healthcare level?	**Reference value (CoVID-19 facility/hospital/quarantine center, Emergency Operation Center (National and Provincial)**								
	* Primary*	0.38	0.15	0.93	0.03	0.46	0.15	1.38	0.17
	* Secondary*	0.46	0.26	0.85	0.01	0.49	0.23	1.01	0.05
	* Tertiary*	0.55	0.33	0.91	0.02	0.46	0.25	0.873	0.01
Perceived professional risk for the next 30 days	**Reference value (High Risk)**								
	* Low risk*	0.95	1.05	0.19	5.764	1.20	0.13	10.92	0.86
	* Medium risk*	0.07	0.24	0.05	1.159	0.18	0.02	1.44	0.10
	* No risk*	0.15	0.33	0.07	1.518	0.23	0.03	1.79	0.16
Perceived Personal risk for the next 30 days	**Reference value (High Risk)**								
	* Low risk*	0.05	0.28	0.07	1.00	0.26	0.05	1.20	0.08
	* Medium risk*	0.04	0.27	0.08	0.97	0.36	0.08	1.65	0.19
	* No risk*	0.23	0.47	0.14	1.60	0.61	0.13	2.72	0.52
Donning Coverall Suit	**Reference value (No)**								
	* Yes*	2.67	1.44	4.96	0.002	0.25	1.73	0.67	4.47
	* I am not sure*	0.92	0.275	3.11	0.900	0.62	0.63	0.10	3.80
Donning Disposable Gown	**Reference value (No)**								
		3.09	1.41	6.73	0.005	0.58	0.70	0.20	2.41
		0.979	0.23	4.10	0.97	0.37	0.36	0.04	3.35
Donning Protecting Googles	**Reference value (No)**								
	Yes	1.709	0.94	3.09	0.07	0.72	0.83	0.31	2.20
Donning Surgical Gloves	**Reference value (No)**								
	Yes	3.245	1.113	9.45	0.03	0.16	2.57	0.67	9.88
Donning Disposable Cover Shoes	**Reference value (No)**								
	Yes	3.09	1.42	6.73	0.004	0.61	1.33	0.42	4.17
Doffing Surgical Masks (N95/99)	**Reference value (No)**								
	Yes	2.20	1.187	4.09	0.01	0.41	1.49	0.56	3.93
Doffing Coverall Suit	**Reference value (No)**								
	Yes	1.84	1.15	2.96	0.01	0.71	0.85	0.37	1.98
Doffing Disposable Gown	**Reference value (No)**								
	Yes	3.76	1.98	7.17	0.001	3.86	1.23	12.08	0.02
Doffing Protecting Googles	**Reference value (No)**								
	Yes	2.09	1.24	3.51	0.005	1.09	0.41	2.87	0.85
Doffing Surgical Gloves	**Reference value (No)**								
	Yes	1.95	1.003	3.807	0.04	0.43	0.13	1.46	0.18
Doffing Disposable Cover Shoes	**Reference value (No)**								
	Yes	2.604	1.458	4.651	0.001	0.622	1.286	0.47	3.49

## Discussion

Before the onset of the COVID-19 pandemic, there was a notable lack of focus on the utilization of personal protective equipment (PPE) among healthcare workers worldwide. While previous research primarily focused on PPE practices in industrial settings for employee safety, studies specifically addressing healthcare workers in low- to low-middle-income countries, particularly the Indian sub-continent were scarce [15–17]. The comprehensive adoption and awareness of PPE kits in healthcare settings have not been extensively observed in recent times [18]. Overcoming these challenges requires the implementation of targeted training programs, along with reinforcing and encouraging adherence to safety measures and hygiene practices. Therefore, this study seeks to bridge this knowledge gap by investigating the impact of pre-pandemic training programs on PPEs on the experiences, preparedness, and practices of healthcare workers in Pakistan during the COVID-19 pandemic.

The findings of our study indicate that the level of training on personal protective equipment (PPE) varied across different healthcare professionals, with doctors exhibiting a higher proportion of training compared to nurses and paramedics. These findings are consistent with prior research highlighting disparities in training levels among various healthcare roles. A study conducted in another low- to low-middle-income country revealed that a significant proportion of doctors (47.3%) and nurses (54.7%) did not receive sufficient training in PPE usage, indicating a higher proportion of nurses lacking training compared to doctors [19]. Another study examining inequalities during the COVID-19 pandemic highlighted that junior staff, such as community health workers, received significantly less training compared to other professions, with only 14.5% of them reporting adequate training in contrast to nearly half of doctors (46%) [20]. Comprehensive and equitable training programs should be implemented to address disparities among healthcare professionals, ensuring all roles, including nurses, paramedics, and junior staff, receive adequate PPE training.

Furthermore, our study uncovered a significant correlation between gender and prior training in the use of Personal Protective Equipment (PPE). A higher proportion of males reported receiving previous training compared to females. This finding aligns with earlier research that has demonstrated variations in training rates based on gender. Notably, a study conducted in England during the peak of the COVID-19 pandemic shed light on the challenges faced by lower-ranking staff, who predominantly consist of females. Their experiences were comparatively more arduous than those of male healthcare workers [21]. Another study delving into gender and racial inequalities amid the COVID-19 crisis unveiled disparities in receiving training for guiding actions. The data showed that 43.9% of white men received training as compared to only 20.94% of black women [20]. Addressing gender inequalities in training on PPE should be prioritized for future crises and pandemics. It is imperative to develop comprehensive strategies that ensure equal access to training opportunities for all healthcare workers, irrespective of gender, to effectively respond to and mitigate the impact of such emergencies.

Moreover, our study demonstrated a significant association between the healthcare level and prior training on PPE. Healthcare staff working at CoVID-19 facilities/hospitals/quarantine centers or tertiary healthcare levels had a higher proportion of prior training. Similar findings were shown by a study conducted in the UK, which uncovered limited accessibility to PPE training among healthcare workers assigned to lower-risk units such as general wards, surgery, and primary care. Media analysis also identified a lack of training for healthcare workers operating in community settings and care homes [22]. Additionally, another study highlighted disparities in conditions between secondary/tertiary healthcare facilities and primary health centers [23]. Possible factors contributing to this discrepancy include greater manpower, increased awareness and demand for PPE, and augmented government funding allocated to secondary and tertiary healthcare facilities [24]. Our findings highlight the alarming lack of attention given to primary care physicians in informational initiatives, leaving them at a significantly higher risk of contracting infections. Given the resource constraints and healthcare infrastructure challenges faced by LMICs, there is a pressing need to prioritize equitable access to PPE training across all levels of care, highlighting the importance of directing greater attention toward these countries.

According to our study, the findings indicate that a larger proportion of healthcare workers who had not received prior training perceived a heightened professional risk for the next 30 days. These findings support prior research indicating that adequate training on PPE usage lowers the risk perception among healthcare workers as compared to those who have not received prior training, as shown in an Italian study [25]. Adequate training is necessary for reducing higher risk perception among healthcare workers to promote a sense of preparedness, confidence, and well-being, ultimately benefiting both the staff and the patients they serve. Additionally, our study found that a higher proportion of participants who received prior training were able to don the coverall suit, compared to those who did not receive prior training. This finding is consistent with existing literature that emphasizes the positive impact of training programs on the practical application of PPE; those who reported having received adequate information during the pandemic reported greater odds of being able to perform the donning procedures [25]. It is crucial to provide comprehensive training programs on the proper use and donning of coverall suits and other personal protective equipment (PPE) for healthcare staff. This will increase their ability to effectively utilize PPE, leading to improved safety, reduced risk of exposure, and better adherence to infection control protocols.

In conclusion, our study contributes to the existing body of literature by highlighting the need for targeted training programs to enhance PPE utilization among healthcare workers. The findings can be generalized to other lower-middle-income countries facing similar challenges. It is crucial to address gender disparities in training, ensure equitable access to training opportunities for all healthcare professionals, and prioritize primary care physicians who may be at a higher risk due to limited training. Integrating PPE training into the medical curriculum can help in establishing a culture of PPE use and improving preparedness for future crises. These efforts are essential in mitigating the impact of emergencies and protecting the well-being of healthcare workers and the communities they serve.

### Clinical significance

The clinical implications of this cross-sectional survey range from improved adherence to PPE protocols and reduced healthcare worker infections to enhanced patient safety, cost savings, knowledge transfer, and the development of evidence-based guidelines. These implications can significantly impact the healthcare system’s response to the COVID-19 pandemic and future infectious disease outbreaks.

### Limitations

The study was conducted online within a relatively short timeframe, but the researchers made efforts to include a substantial number of participants. Due to the lockdown restrictions in the country and the infectious nature of the disease, real-time observation of the participants’ practice of donning and doffing PPE was not conducted which introduces a potential selection bias,

## Conclusion

The result of this study concluded that healthcare levels, type of healthcare, and doffing skills are important predictors of whether healthcare workers have taken prior training in PPE. These findings imply developing effective training programs for healthcare workers to ensure their safety while providing care during pandemics like COVID-19.

### Electronic supplementary material

Below is the link to the electronic supplementary material.

Supplementary Material 1

## Data Availability

All the data generated or analyzed during the current study are available in the supplementary files.
